# JuPOETs: a constrained multiobjective optimization approach to estimate biochemical model ensembles in the Julia programming language

**DOI:** 10.1186/s12918-016-0380-2

**Published:** 2017-01-25

**Authors:** David M. Bassen, Michael Vilkhovoy, Mason Minot, Jonathan T. Butcher, Jeffrey D. Varner

**Affiliations:** 1 Department of Chemical and Biomolecular Engineering, Cornell University, Ithaca, 14853 NY USA; 2 Department of Biomedical Engineering, Cornell University, Ithaca, 14853 NY USA

**Keywords:** Ensemble modeling, Multiobjective optimization, Julia

## Abstract

**Background:**

Ensemble modeling is a promising approach for obtaining robust predictions and coarse grained population behavior in deterministic mathematical models. Ensemble approaches address model uncertainty by using parameter or model families instead of single best-fit parameters or fixed model structures. Parameter ensembles can be selected based upon simulation error, along with other criteria such as diversity or steady-state performance. Simulations using parameter ensembles can estimate confidence intervals on model variables, and robustly constrain model predictions, despite having many poorly constrained parameters.

**Results:**

In this software note, we present a multiobjective based technique to estimate parameter or models ensembles, the Pareto Optimal Ensemble Technique in the Julia programming language (JuPOETs). JuPOETs integrates simulated annealing with Pareto optimality to estimate ensembles on or near the optimal tradeoff surface between competing training objectives. We demonstrate JuPOETs on a suite of multiobjective problems, including test functions with parameter bounds and system constraints as well as for the identification of a proof-of-concept biochemical model with four conflicting training objectives. JuPOETs identified optimal or near optimal solutions approximately six-fold faster than a corresponding implementation in Octave for the suite of test functions. For the proof-of-concept biochemical model, JuPOETs produced an ensemble of parameters that gave both the mean of the training data for conflicting data sets, while simultaneously estimating parameter sets that performed well on each of the individual objective functions.

**Conclusions:**

JuPOETs is a promising approach for the estimation of parameter and model ensembles using multiobjective optimization. JuPOETs can be adapted to solve many problem types, including mixed binary and continuous variable types, bilevel optimization problems and constrained problems without altering the base algorithm. JuPOETs is open source, available under an MIT license, and can be installed using the Julia package manager from the JuPOETs GitHub repository

## Background

Ensemble modeling is a promising approach for obtaining robust predictions and coarse grained population behavior in deterministic mathematical models. It is often not possible to uniquely identify all the parameters in biochemical models, even when given extensive training data [1]. Thus, despite significant advances in standardizing biochemical model identification [2], the problem of estimating model parameters from experimental data remains challenging. Ensemble approaches address parameter uncertainty in systems biology and other fields like weather prediction [3–6] by using parameter families instead of single best-fit parameter sets. Parameter families can be selected based upon simulation error, along with other criteria such as diversity or steady-state performance. Simulations using parameter ensembles can estimate confidence intervals on model variables, and robustly constrain model predictions, despite having many poorly constrained parameters [7, 8]. There are many techniques to generate parameter ensembles. Battogtokh et al., Brown et al., and later Tasseff et al. generated experimentally constrained parameter ensembles using a Metropolis-type random walk [3, 5, 9, 10]. Liao and coworkers developed methods to generate ensembles that all approach the same steady-state, for example one determined by fluxomics measurements [11]. They have used this approach for model reduction [12], strain engineering [13, 14] and to study the robustness of non-native pathways and network failure [15]. Maranas and coworkers have also applied this method to develop a comprehensive kinetic model of bacterial central carbon metabolism, including mutant data [16]. We and others have used ensemble approaches, generated using both sampling and optimization techniques, that have robustly simulated a wide variety of signal transduction processes [9, 10, 17–19], neutrophil trafficking in sepsis [20], patient specific coagulation behavior [21], uncertainty quantification in metabolic kinetic models [22] and to capture cell to cell variation [23]. Further, ensemble approaches have been used in synthetic biology to sample possible biocircuit configurations [24]. Thus, ensemble approaches are widely used to robustly simulate a variety of biochemical systems.

Identification of biochemical models requires significant training data perhaps taken from diverse sources. These real-world data sets often contain intrinsic conflicts resulting from, for example, the use of different cell lines, different measurement technologies, different reagent vendors or lots, uncontrollable experimental artifacts or general cross laboratory variability. Parameter ensembles that optimally balance these inherent conflicts lead to more robust model performance. Multiobjective optimization is an ensemble generation technique that naturally balances conflicts in noisy training data [25]. Multiobjective optimization has been used to identify signal transduction models [18, 23], for the design of synthetic circuits [24], to design the folding behaviors of novel RNAs [26], to design bioprocesses [27], and to understand bacterial adaptation [28]. Thus, it is a widely used approach for a variety of biochemical applications. Previously, we developed the Pareto Optimal Ensemble Technique (POETs) algorithm to address the challenge of competing or conflicting training objectives. POETs, which integrates simulated annealing (SA) and multiobjective optimization through the notion of Pareto rank, estimates parameter ensembles which optimally trade-off between competing (and potentially conflicting) experimental objectives [29]. However, the previous implementation of POETs, in the Octave programming language [30], suffered from poor performance and was not configurable. For example, Octave-POETs does not accommodate user definable objective functions, bounds and problem constraints, cooling schedules, different variable types e.g., a mixture of binary and continuous design variables or custom diversity generation routines. Octave-POETs was also not well integrated into a package or source code management (SCM) system. Thus, upgrades to the approach containing new features, or bug fixes were not centrally managed.

## Implementation

In this software note, we present an open-source implementation of the Pareto optimal ensemble technique in the Julia programming language (JuPOETs). JuPOETs takes advantage of the unique features of Julia to address many of the shortcomings of the previous implementation. Julia is a cross-platform, high-performance programming language for technical computing that has performance comparable to C but with syntax similar to MATLAB/Octave and Python [31]. Julia also offers a sophisticated compiler, distributed parallel execution, numerical accuracy, and an extensive function library. Further, the architecture of JuPOETs takes advantage of the first-class function type in Julia allowing user definable behavior for all key aspects of the algorithm, including objective functions, custom diversity generation logic, linear/non-linear parameter constraints (and parameter bounds constraints) as well as custom cooling schedules. Julia’s ability to naturally call other languages such as Python or C also allows JuPOETs to be used with models implemented in a variety of languages across many platforms. Additionally, Julia offers a built-in package manager which is directly integrated with GitHub, a popular web-based Git repository hosting service offering distributed revision control and source code management. Thus, JuPOETs can be adapted to many problem types, including mixed binary and continuous variable types, bilevel problems and constrained problems without altering the base algorithm, as was required in the previous POETs implementation.

### JuPOETs optimization problem formulation

JuPOETs solves the $\mathcal {K}-$dimensional constrained multiobjective optimization problem:

1 $$ \min_{\mathbf{p}} \left\{ \begin{array}{l} O_{1} \left(\mathbf{x}(t,\mathbf{p}),\mathbf{p} \right) \\ \vdots \\ O_{\mathcal{K}} \left(\mathbf{x}(t,\mathbf{p}),\mathbf{p} \right) \\ \end{array}\right.  $$

subject to the model equations and constraints:

$$\begin{array}{@{}rcl@{}} \mathbf{f} \left(t,\mathbf{x} (t,\mathbf{p}),\dot{\mathbf{x}}(t,\mathbf{p}),\mathbf{u}(t),\mathbf{p}\right) &=&\mathbf{0} \\ g_{1}\left(t,\mathbf{x}(t,\mathbf{p}),\mathbf{u}(t),\mathbf{p}\right) &\geq& 0 \\ &\vdots & \\ g_{\mathcal{C}} \left(t,\mathbf{x}(t,\mathbf{p}),\mathbf{u}(t),\mathbf{p}\right) &\geq& 0  \end{array} $$

and parameter bound constraints:

$$\mathcal{L} \leq \mathbf{p} \leq \mathcal{U} $$

The quantity *O*_*j*_ denotes the *j* ^*th*^ objective function ($j=1,2, \ldots, \mathcal {K}$), typically the sum of squared errors for the *j* ^*th*^ data set for biochemical modeling applications. The terms $\mathbf {f} \left (t,\mathbf {x}(t,\mathbf {p}),\dot {\mathbf {x}}(t,\mathbf {p}),\mathbf {u}(t),\mathbf {p}\right)$ denote the system of model equations (e.g., differential equations, differential algebraic equations or linear/non-linear algebraic equations) where **p** denotes the decision variable vector e.g., unknown model parameters ($\mathcal {D}\times 1$). In typical biochemical modeling applications, the model equations **f**(·) are a system of continuous real-valued non-linear differential equations that comprise a kinetic model, but other types of models e.g., stoichiometric models are also common. The quantity *t* denotes time, **x**(*t*,**p**) denotes the model state (with an initial state **x**_0_), and **u**(*t*) denotes an input vector. The decision variables (e.g., kinetic parameters) can be subject to bounds constraints, where $\mathcal {L}$ and $\mathcal {U}$ denote the lower and upper bounds, respectively as well as $\mathcal {C}$ problem specific constraints $g_{i}\left (t,\mathbf {x}(t,\mathbf {p}),\mathbf {u}(t),\mathbf {p}\right),i=1,\ldots,\mathcal {C}$. The decision variables **p** are typically real-valued kinetic constants, or metabolic fluxes in the case of stoichiometric models. However, other variables types e.g., binary or categorical decision variables can also be accommodated.



**Fig. 1:**
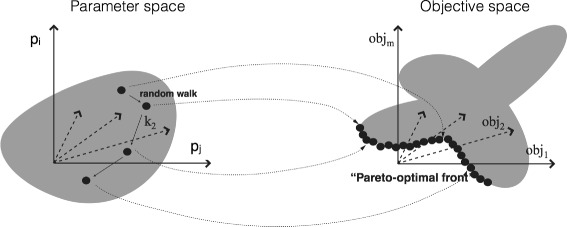
Schematic of multiobjective parameter mapping. The performance of any given parameter set is mapped into an objective space using a ranking function which quantifies the quality of the parameters. The distance away from the optimal tradeoff surface is quantified using the Pareto ranking scheme of Fonseca and Fleming in JuPOETs

JuPOETs integrates simulated annealing (SA) [32] with Pareto ranking to estimate decision variables on or near the optimal tradeoff surface between competing objectives (Fig. 1 and Algorithm 1). A tradeoff surface defines the best possible performance for every conflicting objective, such that an increase in the performance of one objective does not decrease the performance of at least one other objective. Pareto rank is a scalar measure of distance away from the optimal tradeoff surface (low rank is near the surface, while higher ranks are progressively further away). Thus, the central idea underlying POETs is a mapping between the value of the objective vector evaluated at **p**_*i*+1_ (decision variable guess at iteration *i*+1) and the scalar Pareto rank (Fig. 1). Traditional simulated annealing uses a scalar performance value e.g., simulation error to make a probabilistic decision to keep or reject a set of decision variables; decision variables with better performance are always accepted, while those with worse performance are sometimes accepted depending upon a parameter called the temperature. On the other hand, JuPOETs makes this same decision using the Pareto rank instead of a single performance objective. The problem of estimating biochemical model parameters from experimental data is typically posed as an error minimization problem over continuous real-valued decision variables (model parameters) subject to the model equations. A parameter set **p**_*i*+1_ lies along the optimal tradeoff surface if no other parameter guess leads to decreased error for every objective. JuPOETs calculates the performance of a candidate parameter set **p**_*i*+1_ by calling the user defined objective function; objective takes a parameter set as an input, evaluates the model equations, and using this solution, returns the $\mathcal {K}\times {1}$ objective vector. Candidate parameter sets are generated by the user supplied neighbor function; the default implementation of neighbor is a random perturbation, however other perturbation logic can be implemented by the user. The error vector associated with **p**_*i*+1_ is ranked using the builtin Pareto rank function, by comparing the error at iteration *i*+1 to the error archive $\mathcal {O}_{i}$ (all error vectors up to iteration *i* meeting a ranking criterion). Parameter sets on or near the optimal trade-off surface between the objectives have a rank equal to 0 (no other current parameter sets are better). These rank zero parameter sets define the Pareto optimal group for the ensemble, wherein Pareto optimality is defined as a parameter set not being dominated by any other sets within the ensemble. Sets with increasing non-zero rank are progressively further away from the optimal trade-off surface. Thus, a parameter set with a rank =0 is *better* in a trade-off sense than rank >0. We implemented the Fonseca and Fleming ranking scheme in the builtin rank function [33]:

2 $$ \text{\texttt{rank}}\left(\mathcal{O}_{i+1}\left(\mathbf{p}_{i+1}\right)\mid \mathcal{O}_{i}\right)=r  $$

where rank *r* is the number of parameter sets that dominate (are better than) parameter set **p**_*i*+1_, and $\mathcal {O}_{i+1}\left (\mathbf {p}_{i+1}\right)$ denotes the objective vector evaluated at **p**_*i*+1_. We used the Pareto rank to inform the SA calculation. The parameter set **p**_*i*+1_ was accepted or rejected by the SA at each iteration, by calculating an acceptance probability $\mathcal {P}\left (\mathbf {p}_{i+1}\right)$:

3 $$ \mathcal{P}(\mathbf{p}_{i+1}) \equiv \exp{\left\{-\text{\texttt{rank}}\left(\mathcal{O}_{i+1}\left(\mathbf{p}_{i+1}\right) \mid \mathcal{O}_{i} \right)/T \right\}}  $$

where *T* is the simulated annealing temperature; the temperature provides control over how strictly decreasing Pareto rank is enforced. As $\text {\texttt {rank}}\left (\mathcal {O}_{i+1}\left (\mathbf {p}_{i+1}\right)\mid \mathcal {O}_{i}\right)\rightarrow {0}$, the acceptance probability moves toward one, ensuring that we explore parameter sets along the Pareto surface. Occasionally, (depending upon *T*) a parameter set with a high Pareto rank is accepted by the SA allowing a more diverse search of the parameter space. However, as *T* is reduced as a function of iteration count (using the cooling function), the probability of accepting a high-rank set decreases. Parameter sets could also be accepted by the SA but *n* *o* *t* permanently archived in $\mathcal {S}_{i}$, where $\mathcal {S}_{i}$ is the solution archive. Only parameter sets with rank less than or equal to a threshold (rank ≤4 by default) are included in $\mathcal {S}_{i}$, where the archive is re-ranked and filtered after accepting every new parameter set. Parameter bounds were implemented in the neighbor function as box constraints, while problem specific constraints were implemented in objective using a penalty method:

4 $$ {}O_{i}+\lambda \sum\limits_{j=1}^{\mathcal{C}}\min \left\{0,g_{j}\left(t,\mathbf{x}(t,\mathbf{p}),\mathbf{u}(t),\mathbf{p}\right)\right\}\qquad~i=1,\ldots,\mathcal{K}  $$

where *λ* denotes the penalty parameter (*λ*=100 by default). However, because both the neighbor and objective functions are user defined, different constraint implementations are easily defined.

To use JuPOETs, the user specifies the neighbor, acceptance, cooling and objective functions along with an initial decision variable guess. Default implementations of the neighbor, acceptance and cooling functions can be used directly, or they can be overridden by user defined logic. However, the user must provide an implementation of the objective function and provide an initial decision variable guess. Lastly, if the user is operating JuPOETs in hybrid mode, then a refinement function pointer must also be specified. Hybrid mode temporarily switches the search from a multiobjective to a single objective problem, where the sum of the objective functions can be used to update the best (or initial) parameter guess. The specific hybrid mode search logic is up to the user; by default hybrid mode is off, and the default refinement implementation is simply a pass through function. However, we have shown previously that POETs operated in hybrid mode (where the single objective problem used a pattern search approach) had better performance that POETs alone [29]. Thus, hybrid mode is generally recommended for most applications. In addition, there are several user configurable parameters that can be adjusted to control the performance of JuPOETs: maximum_number_of_iterations controls the number of iterations per temperature (default 20); rank_cutoff controls the upper rank bound on the solution archive (default 5); temperature_min controls the minimum temperature after which JuPOETs returns the error and solution archives (default 0.001); show_trace controls the level of output shown to the user (default true). After the completion of the run, JuPOETs returns the parameter solution archive $\mathcal {S}$, objective archive $\mathcal {O}$ and rank archive $\mathcal {R}$. The parameter solution archive $\mathcal {S}$ contains is an $\mathcal {D}\times \mathcal {A}$ array, where $\mathcal {A}$ denotes the number of solutions in the archive when JuPOETs terminated. On the other hand, the objective archive $\mathcal {O}$ is an $\mathcal {K}\times \mathcal {A}$ array containing the performance values for each objective corresponding the columns of $\mathcal {S}$. Lastly, JuPOETs returns the rank archive $\mathcal {R}$ which is an $\mathcal {A}\times {1}$ array of Pareto ranks corresponding to the columns of $\mathcal {S}$. One technical note, if JuPOETs is run from multiple starting locations, and the archives from each of these runs is combined into a single collective archive, the combined parameter rank archive may become invalid. In these cases, it is required to re-rank the parameter sets using the built-in rank function to produce a collective parameter ranking.

## Results and discussion

**Fig. 2:**
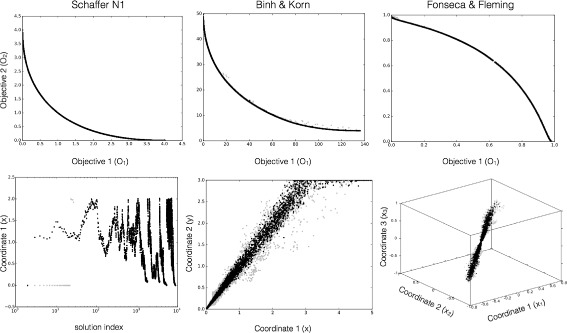
The performance of JuPOETs on the multi-objective test suite. The execution time (wall-clock) for JuPOETs and POETs implemented in Octave was measured for 10 independent trials for the suite of test problems. The number of steps per temperature $\mathcal {I}$ = 10, and the cooling parameter *α* = 0.9 for all cases. The problem domain was partitioned into 10 equal segments, an initial guess was drawn from each segment. For each of the test functions, JuPOETs estimated solutions on (rank zero solutions, *black*) or near (*gray*) the optimal tradeoff surface, subject to bounds and problem constraints

**Fig. 3:**
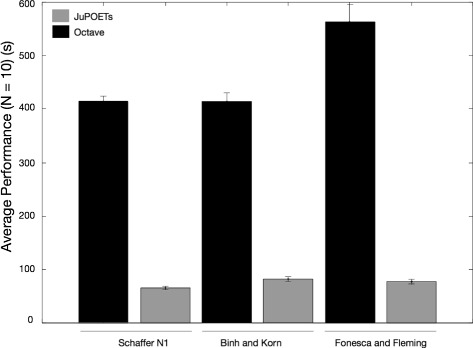
Representative JuPOETs solutions for problems in the multi-objective test suite. The number of steps per temperature $\mathcal {I}$ = 10, and the cooling parameter *α* = 0.9 for all cases. The problem domain was partitioned into 10 equal segments, an initial guess was drawn from each segment. For each of the test functions, JuPOETs estimated solutions on (rank zero solutions, *black*) or near (*gray*) the optimal tradeoff surface, subject to bounds and problem constraints

JuPOETs identified optimal or nearly optimal solutions significantly faster than Octave-POETs for a suite of multiobjective algebraic test problems (Table 1). The algebraic test problems were constrained non-linear functions with bound constraints and additional non-linear constraints on the decision variables in one case. The problems had up to three-dimensional continuous real-valued decision vectors, and each case had two objective functions. The wall-clock time for JuPOETs and Octave-POETs was measured for 10 independent trials for each of the test problems. The same cooling, neighbor, acceptance, and objective logic was employed between the implementations, and all other parameters were held constant. For each test function, the search domain was partitioned into 10 segments, where an initial parameter guess was drawn from each partition. The number of search steps for each temperate was $\mathcal {I}$ = 10 for all cases, and the cooling parameter was *α* = 0.9. On average, JuPOETs identified optimal or near optimal solutions for the suite of test problems six-fold faster (60s versus 400s) than Octave-POETs (Fig. 2). JuPOETs produced the characteristic tradeoff curves for each test problem, given both decision variable bound and problem constraints (Fig. 3). Thus, JuPOETs estimated an ensemble of solutions to constrained multiobjective algebraic test problems significantly faster than the current Octave implementation. Next, we tested JuPOETs on a proof-of-concept biochemical model identification problem.

**Table 1:** Multi-objective optimization test problems. We tested the JuPOETs implementation on three two-dimensional test problems, with one-, two- and three-dimensional parameter vectors. Each problem had parameter bounds constraints, however, on the Binh and Korn function had additional non-linear problem constraints. For the Fonesca and Fleming problem, N = 3

Name	Dimension	Function	Domain	Constraints
Schaffer function	1	*O*_1_(*x*)=*x* ^2^	−10≤*x*≤10	
		*O*_2_(*x*)=(*x*−2)^2^		
Binh and Korn function	2	*O*_1_(*x*,*y*)=4*x* ^2^+4*y* ^2^	0≤*x*≤5	*g*_1_(*x*,*y*)=(*x*−5)^2^+*y* ^2^≤25
		*O*_2_(*x*,*y*)=(*x*−5)^2^+(*y*−5)^2^	0≤*x*≤3	*g*_2_(*x*,*y*)=(*x*−8)^2^+(*y*+3)^2^≤7.7
Fonseca and Fleming function	3	$O_{1}(x_{i})= 1 - \text {exp} \left (- \sum \limits ^{N}_{i= 1} \left (x_{i} - \frac {1}{\sqrt {N}}\right)^{2} \right) $	−4≤*x*_*i*_≤4	
		$O_{2}(x_{i})= 1 - \text {exp} \left (- \sum \limits ^{N}_{i= 1} \left (x_{i} + \frac {1}{\sqrt {N}}\right)^{2} \right) $		

**Fig. 4:**
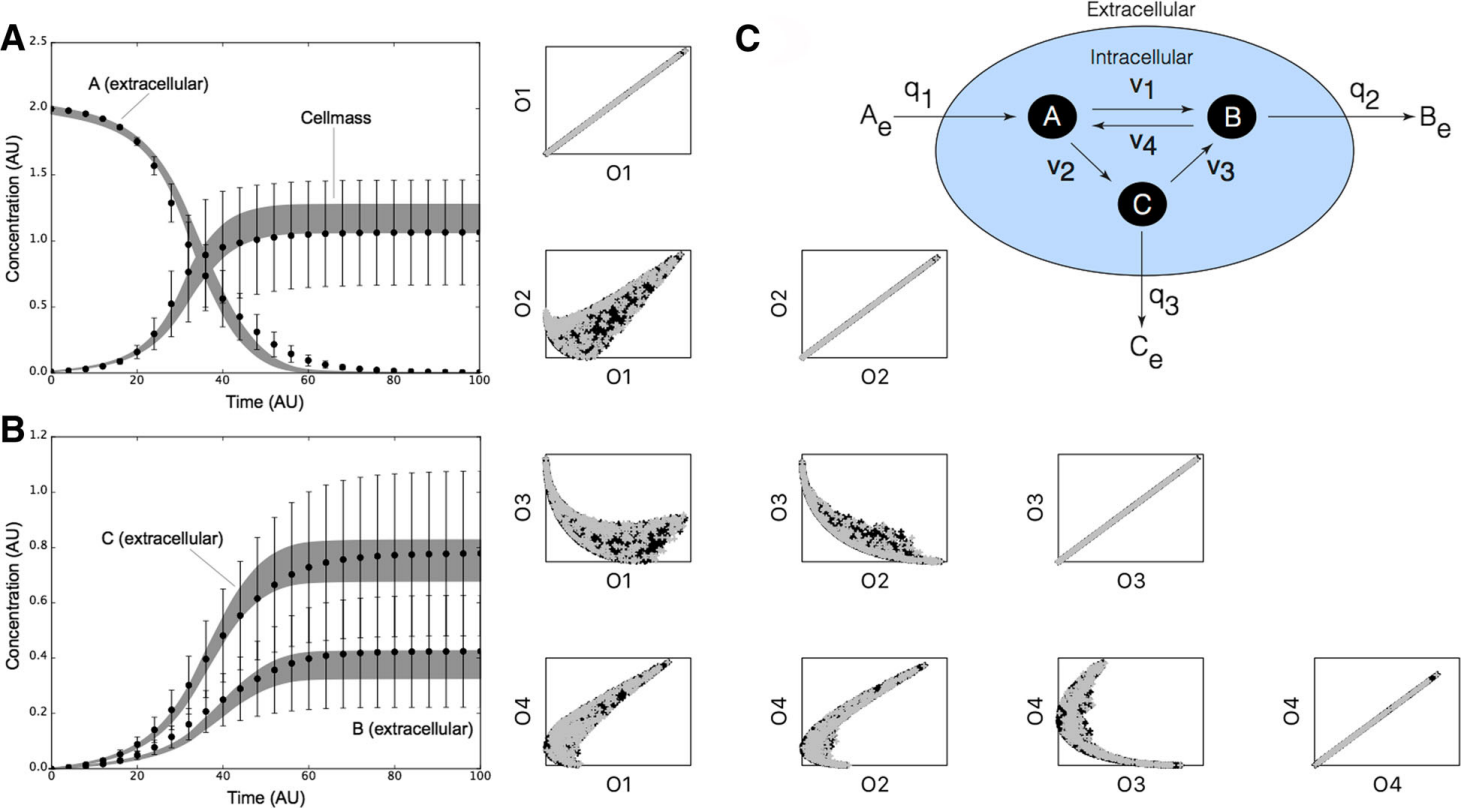
Proof of concept biochemical network study. Inset right: Prototypical biochemical network with six metabolites and seven reactions modeled using the hybrid cybernetic approach (HCM). Intracellular cellmass precursors *A*,*B*, and *C* are balanced (no accumulation) while the extracellular metabolites *A*_*e*_,*B*_*e*_, and *C*_*e*_ are dynamic. The oval denotes the cell boundary, *q*_*j*_ is the *j*th flux across the boundary, and *v*_*k*_ denotes the *k*th intracellular flux. Four data sets (each with *A*_*e*_,*B*_*e*_,*C*_*e*_ and cellmass measurements) were generated by varying the kinetic constants for each biochemical mode. Each data set was a single objective in the JuPOETs procedure. **a** Ensemble simulation of extracellular substrate *A*_*e*_ and cellmass versus time. **b** Ensemble simulation of extracellular substrate *B*_*e*_ and *C*_*e*_ versus time. The gray region denotes the 95% confidence estimate of the mean ensemble simulation. The data points denote mean synthetic measurements, while the error bars denote the 95% confidence estimate of the measurement computed over the four training data sets. **c** Trade-off plots between the four training objectives. The quantity *O*_*j*_ denotes the jth training objective. Each point represents a member of the parameter ensemble, where *gray* denotes rank 0 sets, while *black* denotes rank 1 sets. Ensembles were generated using POETs without employing local refinement

JuPOETs estimated an ensemble of biochemical model parameters that were consistent with the mean of synthetic training data (Fig. 4). Four synthetic training data sets were generated from a prototypical biochemical network consisting of 6 metabolites and 7 reactions (Fig. 4, inset right). We considered a common case in which the same extracellular measurements of *A*_*e*_,*B*_*e*_,*C*_*e*_ and cellmass were made on four hypothetical cell types, each having the same biological connectivity but different performance. Network dynamics were modeled using the hybrid cybernetic model with elementary modes (HCM) approach of Ramkrishna and coworkers [34]. In the HCM approach, metabolic networks are first decomposed into a set of elementary modes (EMs) (chemically balanced steady-state pathways, see [35]). Dynamic combinations of elementary modes are then used to characterize network behavior. Each elementary mode is catalyzed by a pseudo enzyme; thus, each mode has both kinetic and enzyme synthesis parameters. The proof of concept network generated 6 EMs, resulting in 13 model parameters (continuos real-valued decision variables). The synthetic training data was generated by randomly varying these parameters.

The general form of the biochemical test problem was given by:

5 $$ \min_{\mathbf{p}}\left(O_{1},\ldots,O_{\mathcal{K}}\right)  $$

subject to model and bounds constraints. We considered four training data sets ($\mathcal {K}=4$), each of which contained time-series measurements of *A*_*e*_,*B*_*e*_,*C*_*e*_ and cellmass. Each objective $O_{j},~j=1,\ldots,\mathcal {K}$ quantified the squared difference between the simulated (*x*_*i*_) and measured extracellular species abundance (*y*_*i*_) in the *j* ^*th*^ data set:

6 $$ O_{j} = \sum_{i} \sum_{\tau} \left(x_{i}(\tau)-y_{i}(\tau)\right)^{2}\qquad j=1,\ldots,\mathcal{K}  $$

where, *i* denotes the species index and *τ* denotes the time index. The abundance of extracellular species *i* (*x*_*i*_), the pseudo enzyme *e*_*l*_ (catalyzes flux through mode *l*), and cellmass were governed by the model equations:

$$\begin{array}{@{}rcl@{}} \frac{dx_{i}}{dt} & = & \sum\limits_{j = 1}^{\mathcal{R}} \sum\limits_{l=1}^{\mathcal{L}} \sigma_{ij} z_{jl} q_{l} \left(\mathbf{e},\mathbf{p},\mathbf{x}\right)c \qquad {i=1,\ldots,\mathcal{M}} \\[-4.5pt] \frac{de_{l}}{dt} & = & \alpha_{l} + r_{El}\left(\mathbf{p},\mathbf{x}\right)u_{l} - \left(\beta_{l}+r_{G}\right)e_{l} \qquad l=1,\ldots,\mathcal{L} \\[-4.5pt] \frac{dc}{dt} & = & r_{G}c  \end{array} $$

where $\mathcal {R}$ and $\mathcal {M}$ denote the number of reactions and extracellular species in the model and $\mathcal {L}$ denotes the number of elementary modes. The quantity *σ*_*ij*_ denotes the stoichiometric coefficient for species *i* in reaction *j* and *z*_*jl*_ denotes the normalized flux for reaction *j* in mode *l*. If *σ*_*ij*_>0, species *i* is produced by reaction *j*; if *σ*_*ij*_<0, species *i* is consumed by reaction *j*; if *σ*_*ij*_=0, species *i* is not connected with reaction *j*. Extracellular species, cellmass and pseudo-enzyme were subject to the initial conditions **x**(*t*_*o*_)=**x**_*o*_, *c*(*t*_*o*_)=*c*_*o*_ and *e*_*l*_=0.5, respectively. The term *q*_*l*_(**e**,**p**,**x**) denotes the specific uptake/secretion rate for mode *l* where **e** denotes the pseudo enzyme vector, **p** denotes the unknown kinetic parameter vector (decision variables), **x** denotes the extracellular species vector, and *c* denotes the cell mass; *q*_*l*_(**e**,**p**,**x**) is the product of a kinetic term ($\bar {q}_{l}$) and a control variable governing enzyme activity. Flux through each mode was catalyzed by a pseudo enzyme *e*_*l*_, synthesized at the regulated specific rate *r*_*E*,*l*_(**p**,**x**), and constitutively at the rate *α*_*l*_. The term *u*_*l*_ denotes the cybernetic variable controlling the synthesis of enzyme *l*. The term *β*_*l*_ denotes the rate constant governing non-specific enzyme degradation, and *r*_*G*_ denotes the specific growth rate through all modes. The specific uptake/secretion rates and the specific rate of enzyme synthesis were modeled using saturation kinetics. The specific growth rate was given by:

$$ r_{G} = \sum\limits_{l = 1}^{\mathcal{L}}z_{\mu l}q_{l}\left(\mathbf{e},\mathbf{p},\mathbf{x}\right) $$

where *z*_*μl*_ denotes the growth flux *μ* through mode *l*. The control variables *u*_*l*_ and *v*_*l*_, which control the synthesis and activity of each enzyme respectively, were given by:

7 $$ u_{l} = \frac{z_{sl}\bar{q}_{l}}{\sum\limits_{l = 1}^{\mathcal{L}}z_{sl}\bar{q}_{l}}  $$

and

8 $$ v_{l} = \frac{z_{sl}\bar{q}_{l}}{\max\limits_{l=1,\ldots,\mathcal{L}}z_{sl}\bar{q}_{l}}  $$

where *z*_*sl*_ denotes the uptake flux of substrate *s* through mode *l*. Each unknown kinetic parameter was continuous and real-valued, and subject to bounds constraints: $\mathcal {L} \leq \mathbf {p} \leq \mathcal {U}$.

**Fig. 5:**
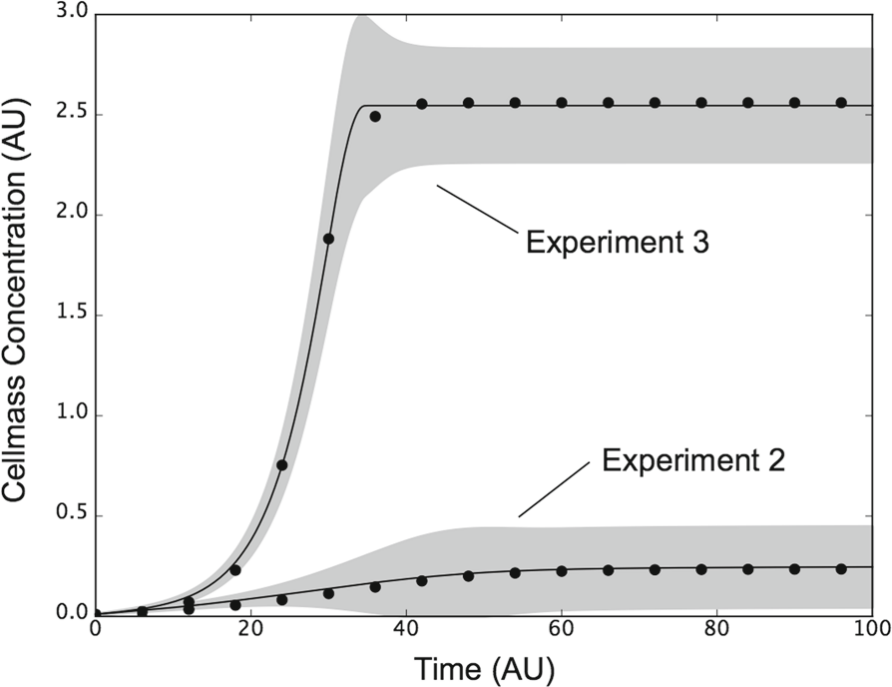
Experiment to experiment variation captured by the ensemble. Cellmass measurements (*points*) versus time for experiment 2 and 3 were compared with ensemble simulations. The full ensemble was sorted by simultaneously selecting the top 25% of solutions for each objective with rank ≤ 1. The best fit solution for each objective (*line*) ± 1-standard deviation (*gray region*) for experiment 2 and 3 brackets the training data despite significant differences the training values between the two data sets

JuPOETs produced an ensemble of approximately $\dim {\mathcal {S}}\simeq $ 13,000 parameter sets that captured the mean of the measured data sets for extracellular metabolites and cellmass (Fig. 4 a and b). JuPOETs minimized the difference between the simulated and measured values for extracellular metabolites A _*e*_, B _*e*_, C _*e*_ and cellmass, where the residual for each data set was treated as a single objective (leading to four objectives). The 95% confidence estimate produced by the ensemble was consistent with the mean of the measured data, despite having significant uncertainty in the training data. JuPOETs produced a consensus estimate of the synthetic data by calculating optimal trade-offs between the training data sets (Fig. 4 c). Multiple trade-off fronts were visible in the objective plots, for example between data set 3 (O_3_) and data set 2 (O_2_). Thus, without a multiobjective approach, it would be challenging to capture these data sets as fitting one leads to decreased performance on the other. However, the ensemble contained parameter sets that described each data set independently (Fig. 5). Thus, JuPOETs produced an ensemble of parameters that gave the mean of the training data for conflicting data sets, while simultaneously estimating parameter sets that performed well on each individual objective function.

Currently, JuPOETs does not consider parameter identifiability when constructing parameter ensembles. Although JuPOETs produces parameter estimates that give model performance similar to the training data, we do not have strict statistical confidence that the *true* parameter values are contained within the ensemble. However, despite this, ensembles produced by POETs can be predictive [18, 23]. Thus, JuPOETs produces a collection of parameters that are constrained by the performance of the model, and not by specific hypotheses regarding the individual values of the raw model parameters. Of course, knowledge of specific parameter values, or the relationship between parameter combinations, can be used to inform the search through either bounds or problem specific constraints (for example, as demonstrated in the first example problem).

## Conclusions

In this software note, we presented JuPOETs, a multiobjective technique to estimate parameter ensembles in the Julia programming language. JuPOETs is open source, and available for download under an MIT license from the JuPOETs GitHub repository at https://github.com/varnerlab/POETs.jl. We demonstrated JuPOETs on a suite of algebraic test problems, and a proof-of-concept ODE based biochemical model. While JuPOETs outperformed (and was significantly more flexible) than the previous Octave implementation, there are several areas that could be explored further. First, JuPOETs should be compared with other multiobjective evolutionary algorithms (MOEAs) to determine its relative performance on test and real world problems. Many evolutionary approaches e.g., the non-dominated sorting genetic algorithm (NSGA) family of algorithms, have been adapted to solve multiobjective problems [36, 37]. However, since there is a lack of open source Julia implementations of these alternative approaches, we did not benchmark the relative performance of JuPOETs in this note. One advantage that JuPOETs may have when compared to a strictly evolutionary approaches, is the inclusion of a local refinement step (hybrid mode), which temporarily reduces the problem to a single objective formulation. Previously, POETs run in hybrid mode led to better convergence on a proof-of-concept signal transduction model compared to the same approach without the hybrid refinement step [29]. Other hybrid multiobjective methods have also been shown to be more efficient than evolutionary approaches alone, for a variety of biochemical optimization problems [24, 38]. Thus, there are several different algorithms that we can use to benchmark, and improve the performance of JuPOETs, after we implement them in Julia. Another strategy to improve the performance of JuPOETs is to reduce the number (or cost) of function evaluations that are required to obtain optimal or near optimal solutions. For example, in many real world parameter estimation problems, the bulk of the execution time is spent evaluating the objective functions. One strategy to improve JuPOETs performance could be to optimize surrogates [39], while another would be parallel execution of the objective functions. Currently, JuPOETs serially evaluates the objective function vector. However, parallel evaluation of the objective functions e.g., using the parallel Julia macro or other techniques, could be implemented without significantly changing the JuPOETs run loop. Taken together, JuPOETs demonstrated improved flexibility, and performance over POETs in parameter identification and ensemble generation for multiple objectives. JuPOETs has the potential for widespread use due to the flexibility of the implementation, and the high level syntax and distribution tools native to the Julia programming language.

## Availability and requirements

JuPOETs is open source, available under an MIT software license. The JuPOETs source code is freely available from the JuPOETs GitHub repository at https://github.com/varnerlab/POETs.jl. All samples used in this study are included in the sample/biochemical and sample/test_functions subdirectories of the JuPOETs GitHub repository. JuPOETs can be run on all common.**Operating system environments:** (Linux, Mac OS, Windows).
